# Supplementary figures and images for: Dopamine negatively modulates the NCA ion channels in C. elegans
Source: PLoS Genet. 2017 Oct 2;13(10):e1007032. doi: 10.1371/journal.pgen.1007032 (PMC5638609; doi:10.1371/journal.pgen.1007032)

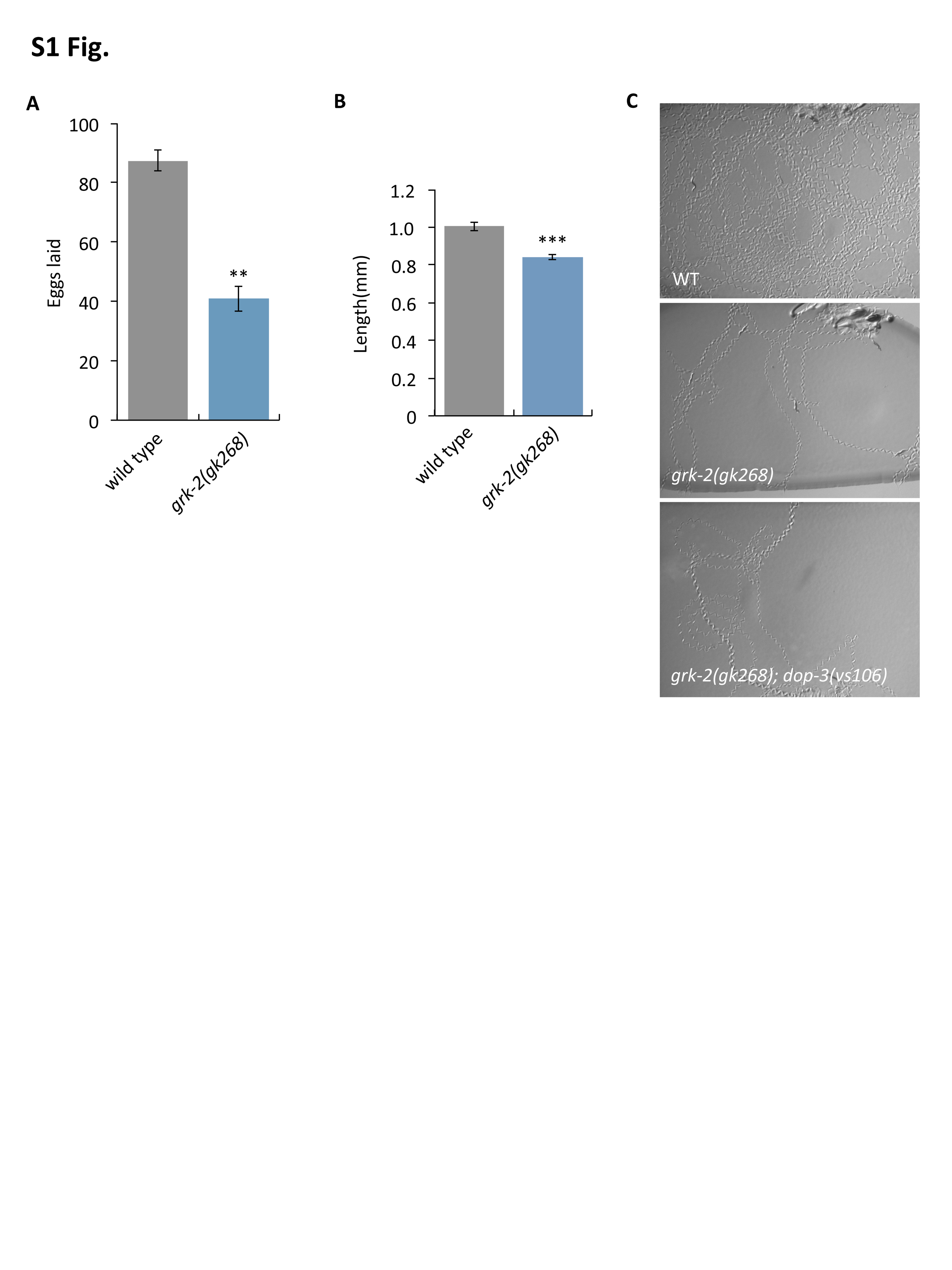

Supplement: S1 Fig — (A) The grk-2(gk268) mutant has an egg-laying defect. The graph shows the number of eggs laid by 5 animals in a 2 h period. (**, P<0.01. Error bars = SEM; n = 2 plates of 5 animals each). (B) The grk-2(gk268) mutant animals have short bodies. (***, P<0.001. Error bars = SEM; n = 10).(C) A dop-3 mutation does not suppress the restricted exploration behavior of grk-2 mutants. Shown are images of tracks of five wild-type, grk-2(gk268), and grk-2(gk268); dop-3(vs106) mutant animals that were allowed to explore a bacterial lawn for 2 hours at room temperature. (TIF) [file pgen.1007032.s001.tif]

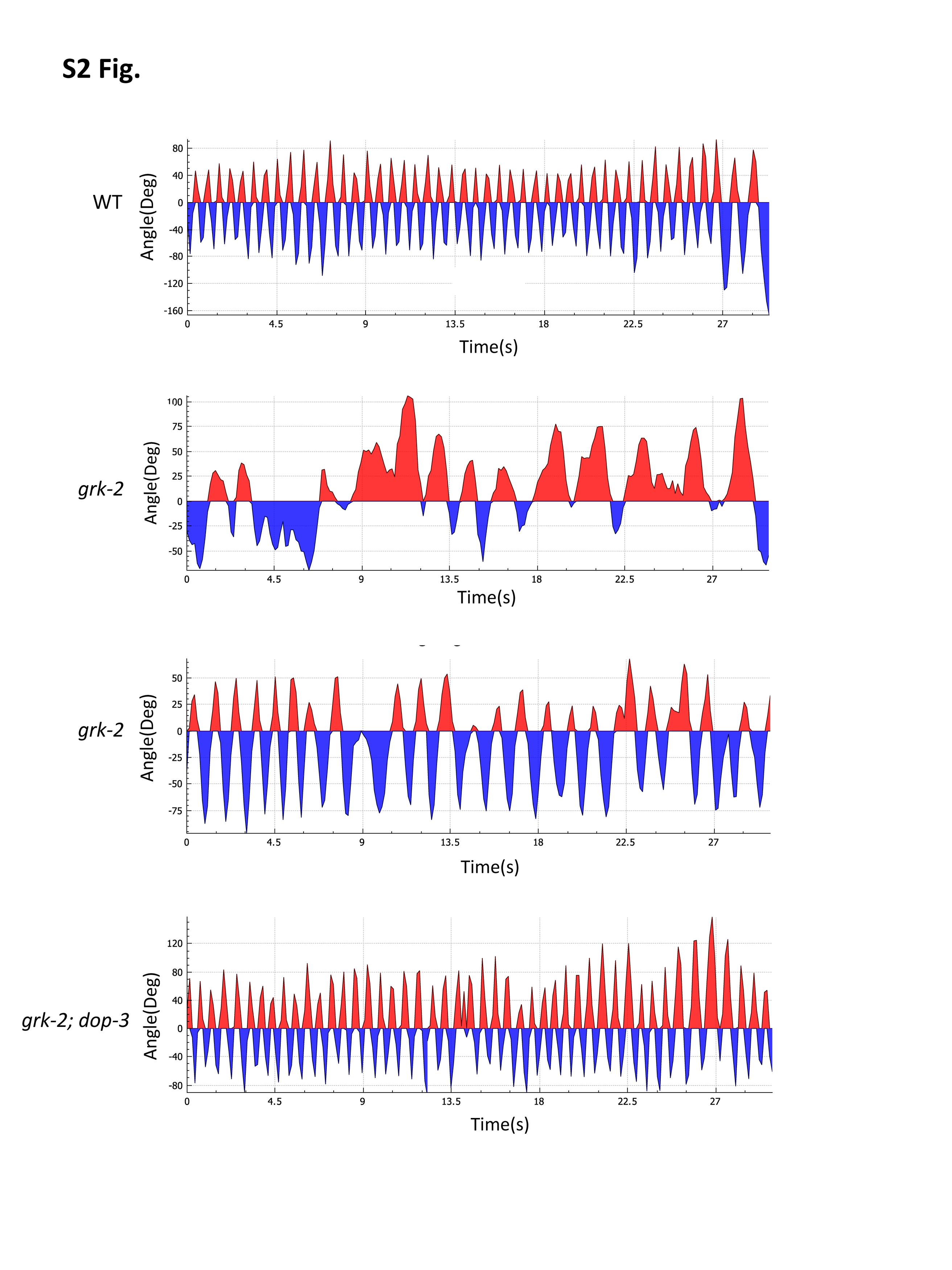

Supplement: S2 Fig — Shown are plots of bending angle (midpoint) versus time for representative individual animals. The two plots of grk-2(gk268) mutant animals show individuals with strong and weak swimming defects. The dop-3(vs106) mutation suppresses the swimming defects of the grk-2(gk268) mutant. (TIF) [file pgen.1007032.s002.tif]

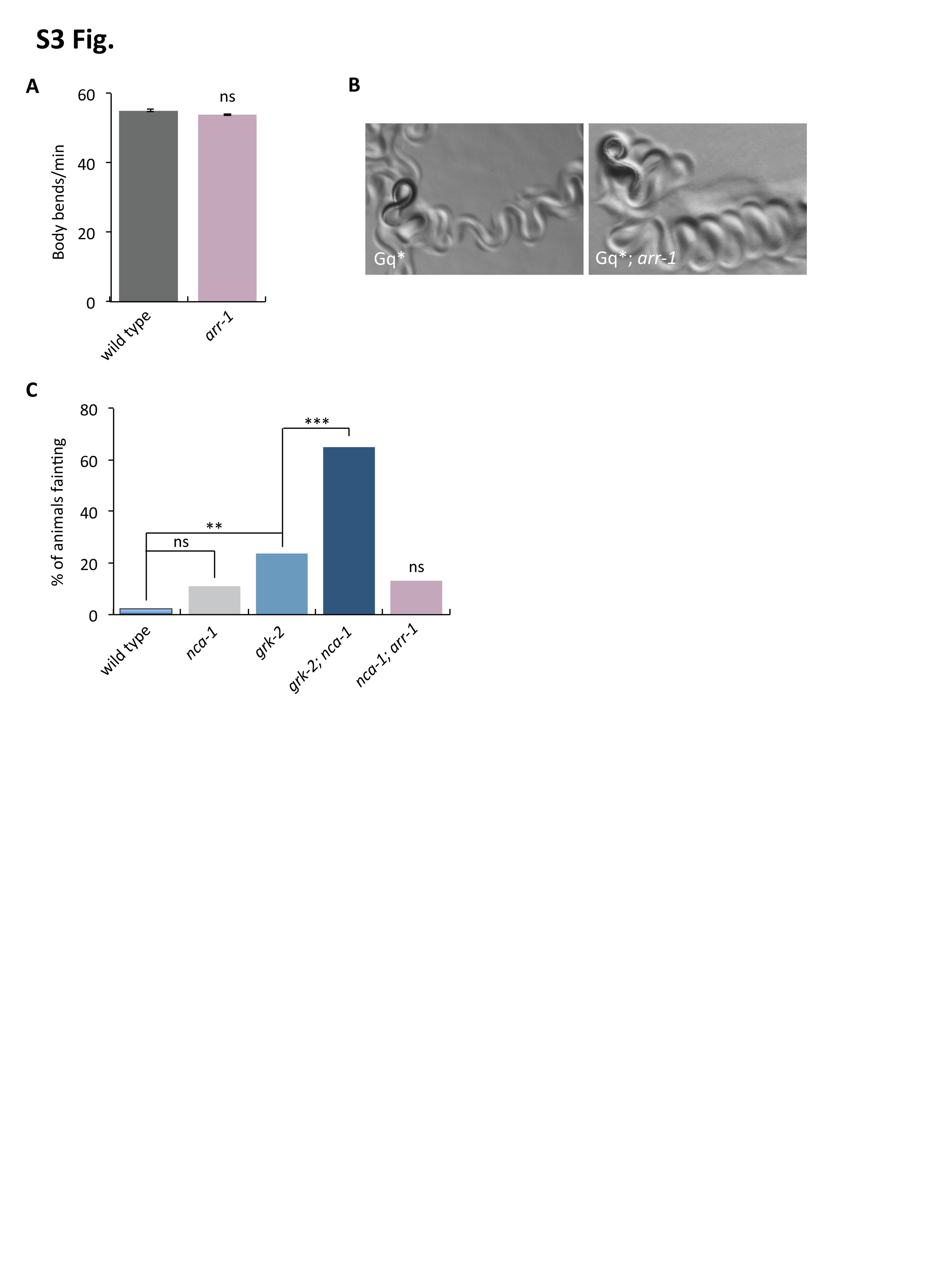

Supplement: S3 Fig — (A) The arr-1(ok401) mutant has no locomotion defect. (ns, P>0.05. Error bars = SEM; n = 10). (B) The arr-1(ok401) mutation does not suppress the loopy posture of the egl-30(tg26) mutant. (C) The arr-1(ok401) mutation, in contrast to a grk-2(gk268) mutation, does not cause a fainting phenotype in an nca-1(gk9) mutant background. Shown is the percentage of animals that faint when moving backwards. The wild-type, nca-1, grk-2, and grk-2; nca-1 data are the same data shown in S6F Fig. The graph shows the combined data from two independent experiments, each with n = 20–40. (**, P<0.01; ***, P<0.001; ns, P>0.05). (TIF) [file pgen.1007032.s003.tif]

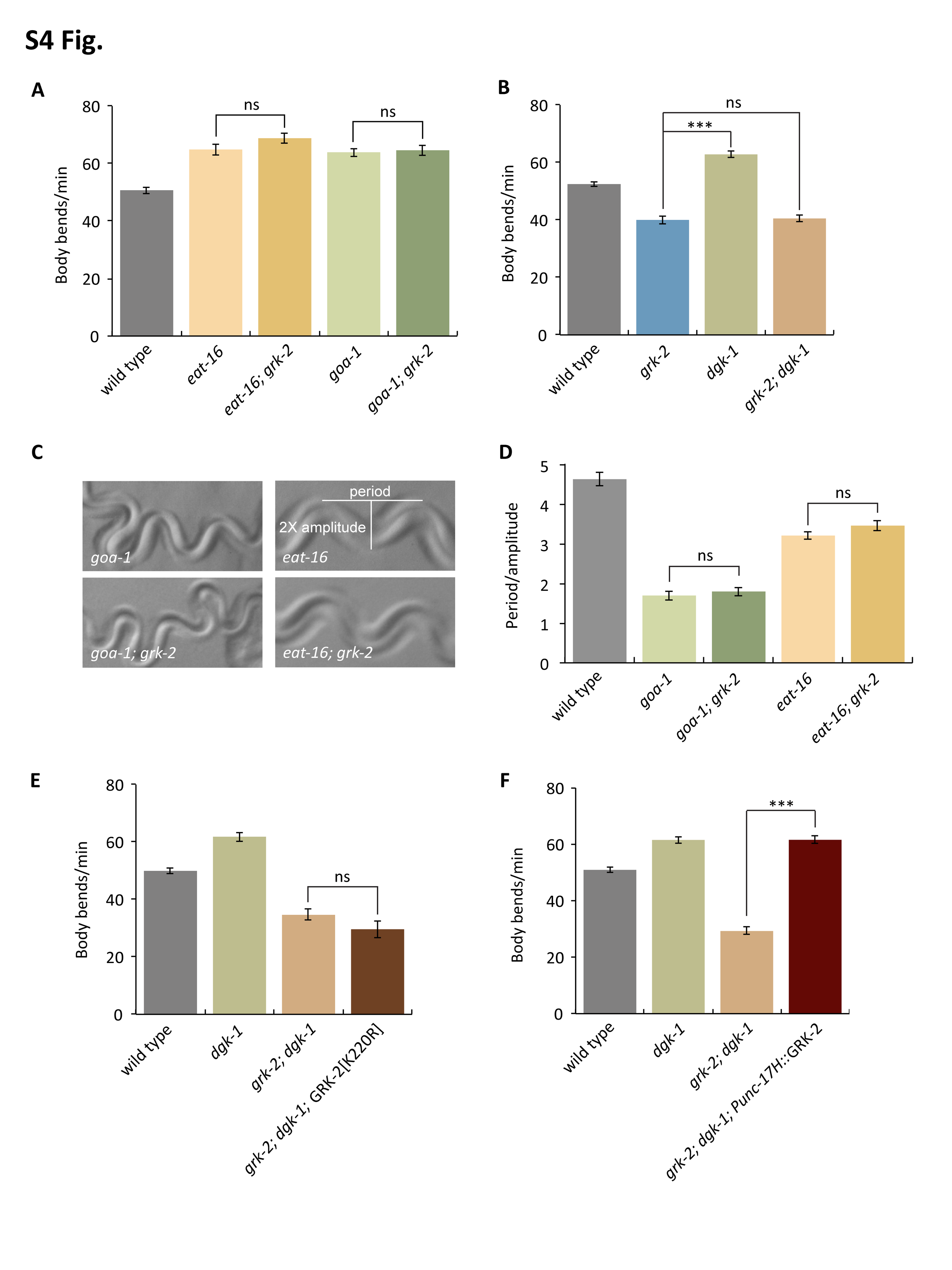

Supplement: S4 Fig — (A) The grk-2(gk268) mutation does not suppress the hyperactive locomotion of the eat-16(tm775) and goa-1(sa734) mutants. (ns, P>0.05. Error bars = SEM; n = 10–20). (B) The grk-2(gk268) mutation suppresses the hyperactive locomotion phenotype of the dgk-1(sy428) mutant. (***, P<0.001. ns, P>0.05. Error bars = SEM; n = 10–20). (C-D) The grk-2(gk268) mutation does not suppress the loopy posture of the eat-16(tm775) and goa-1(sa734) mutants. (ns, P>0.05. Error bars = SEM; n = 5). (E) The kinase-dead GRK-2 does not reverse the grk-2 suppression of the dgk-1 hyperactive locomotion phenotype. Expression of the kinase-dead GRK-2[K220R] mutant under its own promoter (transgene yakEx48) does not reverse the grk-2 suppression of dgk-1 hyperactivity. (ns, P>0.05. Error bars = SEM; n = 10–20). (F) Expression of the grk-2 cDNA under a head acetylcholine neuron promoter (transgene yakEx51) reverses the grk-2 suppression of the hyperactive locomotion of the dgk-1(sy428) mutant. (***, P<0.001. Error bars = SEM; n = 10–20). (TIF) [file pgen.1007032.s004.tif]

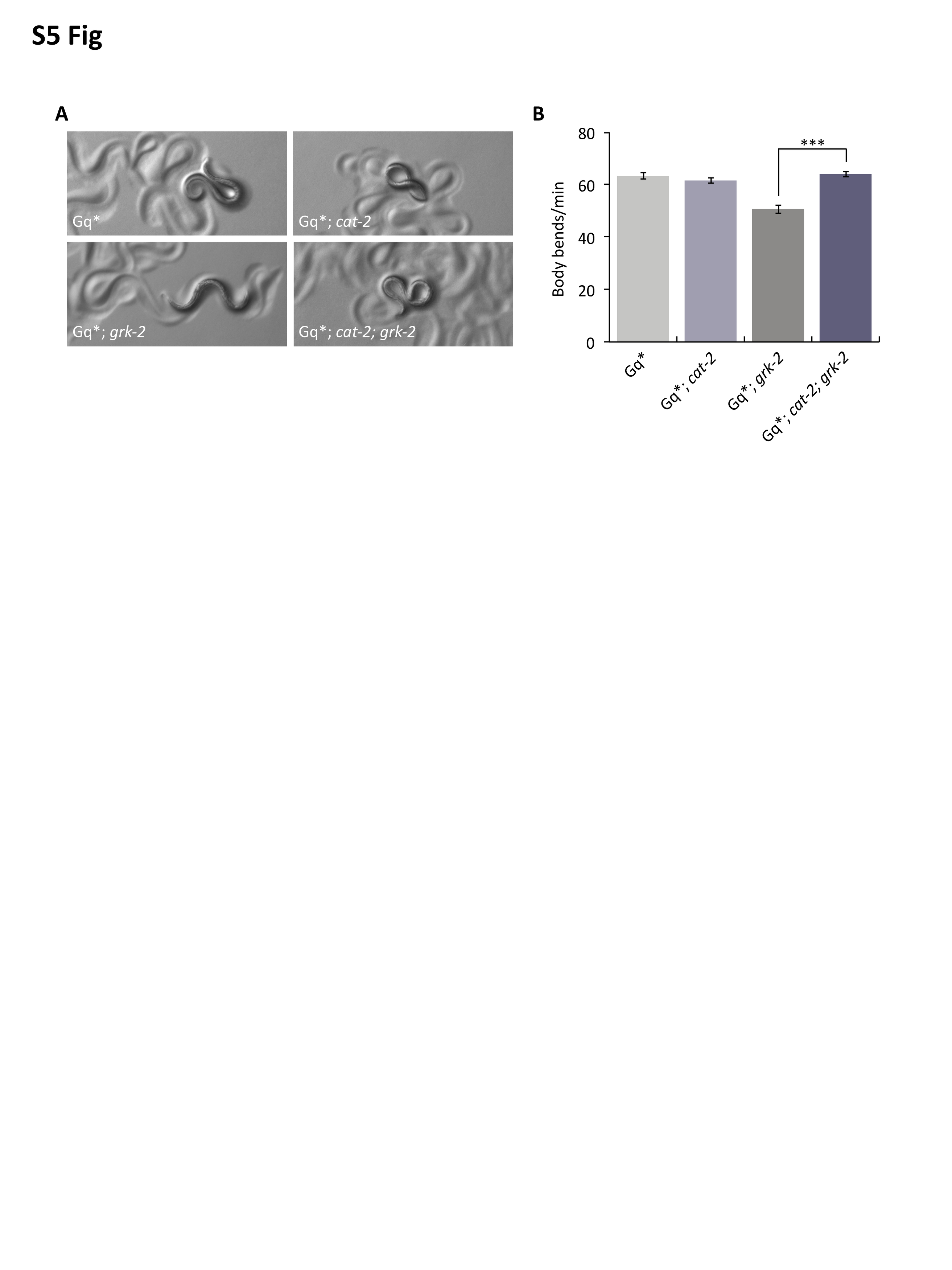

Supplement: S5 Fig — The grk-2(gk268) mutation suppresses the loopy posture and hyperactive locomotion of the activated Gq mutant egl-30(tg26) (Gq*). The cat-2(e1112) mutation reverses the grk-2 suppression of the loopy posture (A) and hyperactive locomotion (B) of Gq*. (***, P<0.001. Error bars = SEM; n = 15–20). (TIF) [file pgen.1007032.s005.tif]

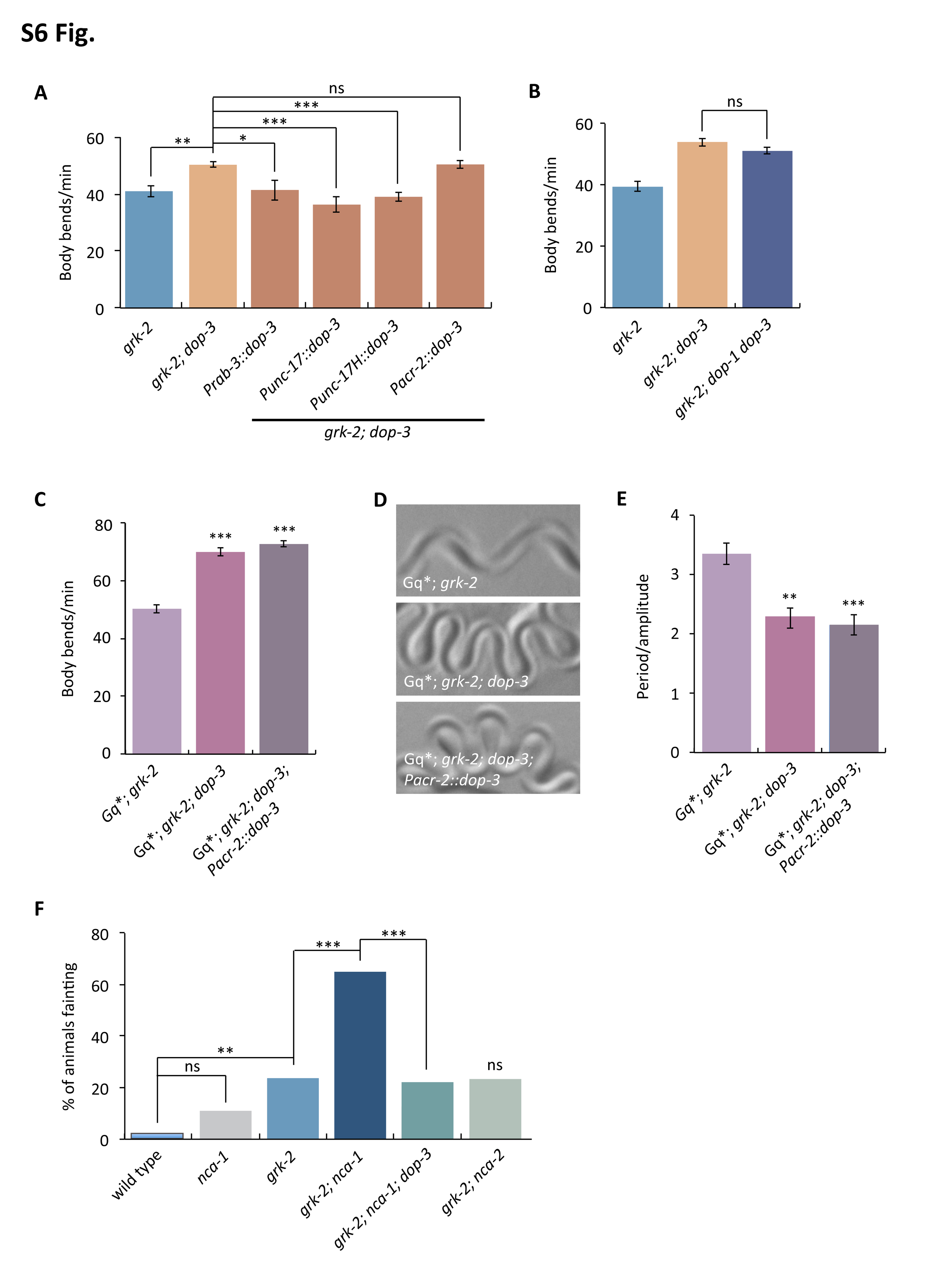

Supplement: S6 Fig — (A) The dop-3 suppression of grk-2 is reversed by dop-3 expression in head acetylcholine neurons. The dop-3 cDNA was expressed in the grk-2(gk268); dop-3(vs106) double mutant under a pan-neuronal promoter (Prab-3, transgene yakEx112), acetylcholine neuron promoter (Punc-17, transgene yakEx111), head acetylcholine neuron promoter (Punc-17H, transgene yakEx110) and ventral cord acetylcholine motor neuron promoter (Pacr-2, transgene yakEx109). Expression of dop-3 driven by the pan-neuronal, acetylcholine neuron, and head acetylcholine neuron promoters reversed the dop-3(vs106) mutant suppression of the slow locomotion of grk-2(gk268) mutant animals. (*, P<0.05; **, P<0.01; ***, P<0.001; ns, P>0.05. Error bars = SEM; n = 10–33). (B) A dop-1 mutation does not affect the dop-3 suppression of the grk-2 slow locomotion phenotype. grk-2; dop-3 mutants move more rapidly than the grk-2 mutant. The dop-1(vs100) mutation does not affect grk-2(gk268); dop-3(vs106) locomotion. (ns, P>0.05. Error bars = SEM; n = 23–34). (C-E) Expression of dop-3 in ventral cord motor neurons is not sufficient to reverse the hyperactive locomotion and loopy posture of egl-30(tg26); grk-2; dop-3 mutant animals. (C) Expression of dop-3 driven by the ventral cord neuron promoter (Pacr-2, transgene yakEx109) does not reduce the hyperactivity of egl-30(tg26); grk-2(gk268); dop-3(vs106) mutant animals. (***, P<0.001; ns, P>0.05. Error bars = SEM; n = 10). (D-E) Expression of dop-3 driven by the ventral cord neuron promoter (Pacr-2, transgene yakEx109) does not reverse the loopy waveform of egl-30(tg26); grk-2(gk268); dop-3(vs106) mutant animals. (**, P<0.01; ***, P<0.001; ns, P>0.05. Error bars = SEM; n = 10). (F) A dop-3 mutation suppresses the fainting phenotype of grk-2; nca-1 mutants. Shown is the percentage of animals that faint when moving backwards. The wild-type, nca-1, grk-2, and grk-2; nca-1 data are the same data shown in S3C Fig. The graph shows the combined data from two independent experimen [file pgen.1007032.s006.tif]

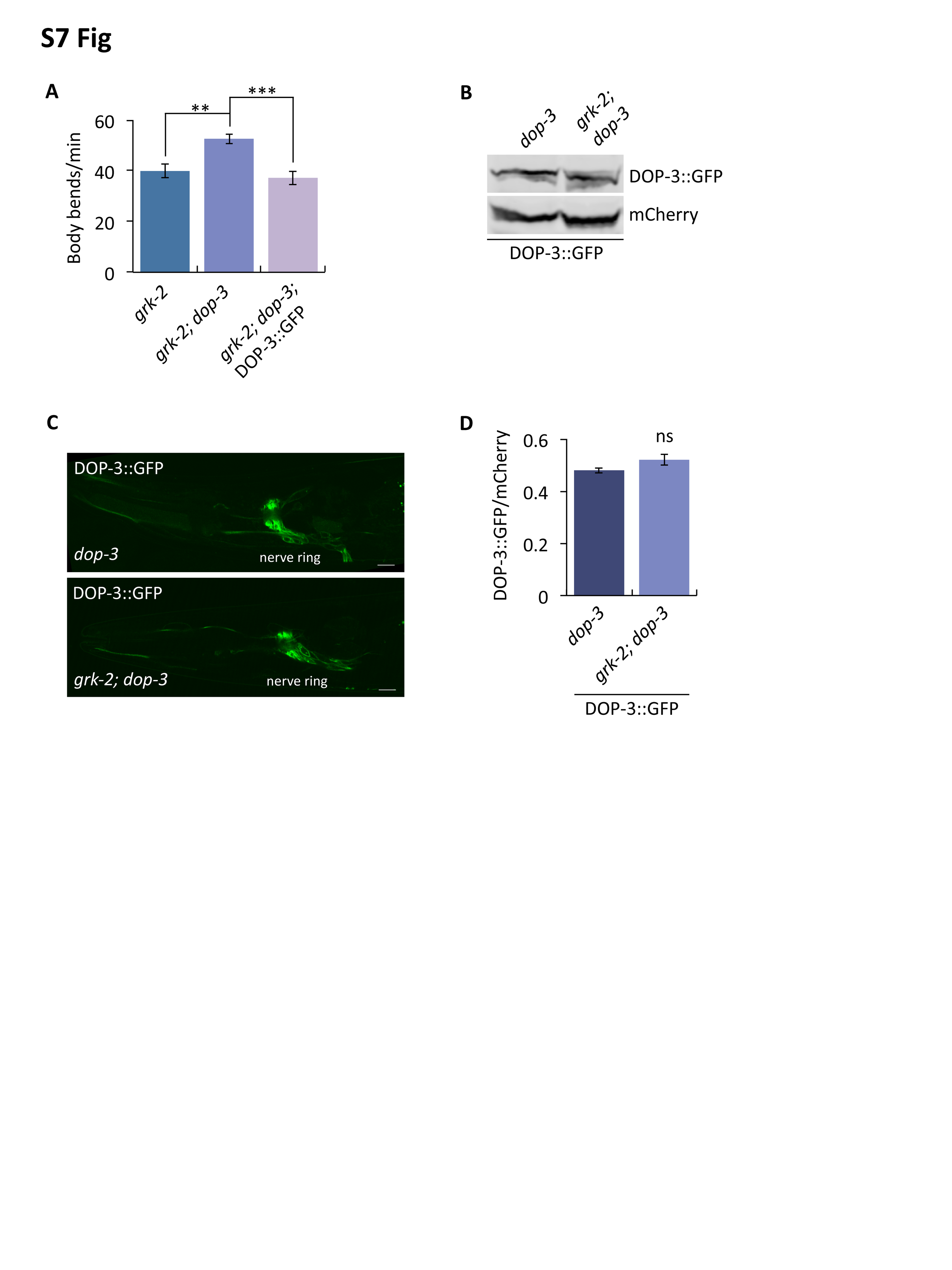

Supplement: S7 Fig — (A) DOP-3::GFP expression driven by the grk-2 promoter (transgene yakEx130) reverses the dop-3 mutant suppression of the slow locomotion phenotype of grk-2 mutants. (**, P<0.01; ***, P<0.001. Error bars = SEM; n = 10). (B) DOP-3::GFP levels remain unaffected in grk-2 mutants. Immunoblot of extracts derived from dop-3 or grk-2; dop-3 animals expressing Pgrk-2::DOP-3::GFP and Pmyo-2::mCherry from an extrachromosomal array (transgene yakEx130). The experiment was repeated twice with similar results. (C,D) DOP-3::GFP subcellular localization and level of expression remain unaffected in grk-2 mutants. (C) Representative images of a Z-stack projection of the area around the nerve ring in the head of dop-3 or grk-2; dop-3 mutant animals expressing Pgrk-2::DOP-3::GFP (transgene yakEx130). (D) Quantification of the ratio of DOP-3::GFP to mCherry in the region around the nerve ring of dop-3 or grk-2; dop-3 animals expressing Pgrk-2::DOP-3::GFP and Pmyo-2::mCherry (transgene yakEx130). (ns, P>0.05. Error bars = SEM; n = 10). (TIF) [file pgen.1007032.s007.tif]

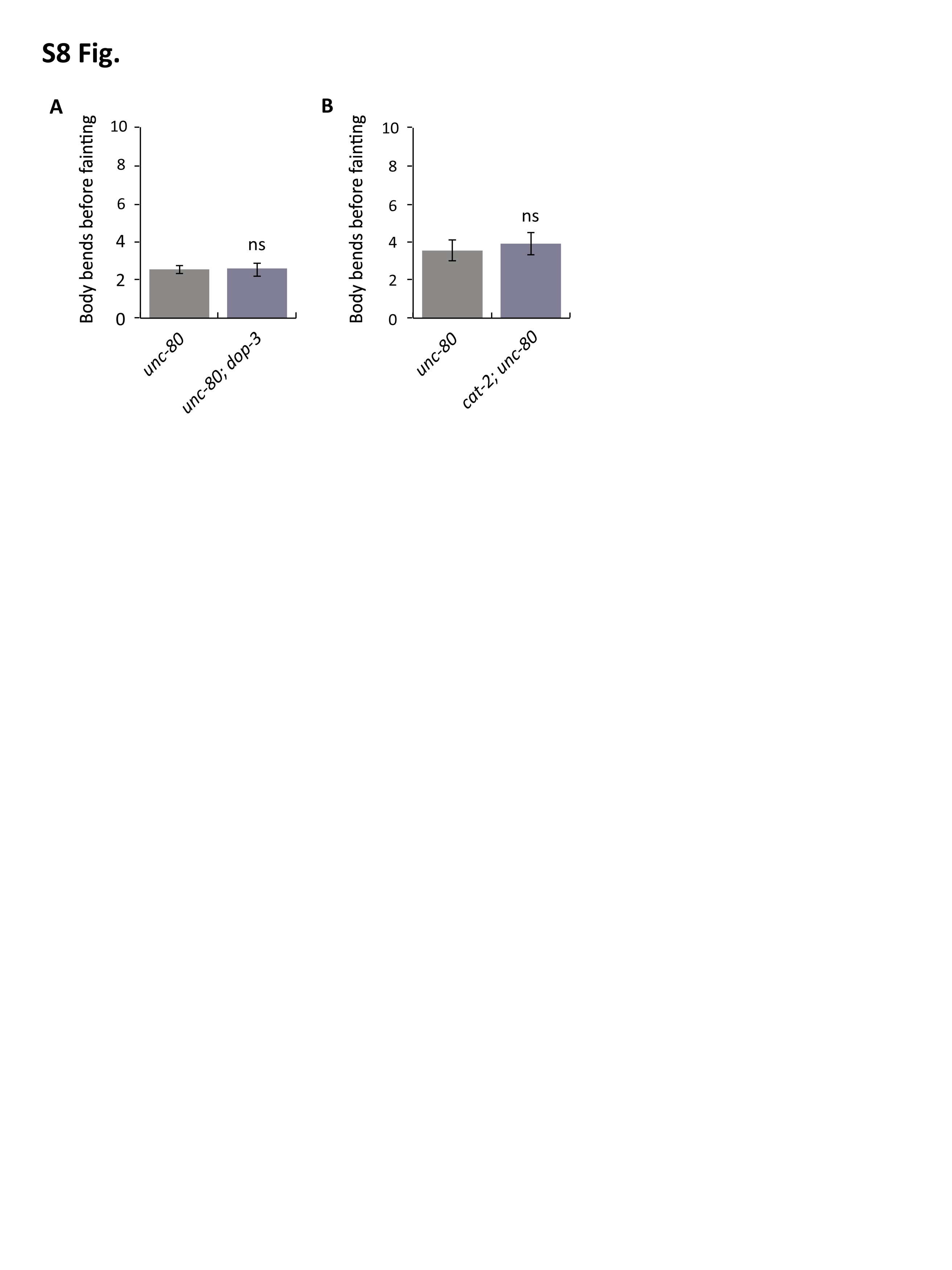

Supplement: S8 Fig — (A) The dop-3(vs106) mutation does not suppress the strong forward fainting phenotype of the unc-80(ox330) mutant. (ns, P>0.05. Error bars = SEM; n = 20). (B) The cat-2(e1112) mutation does not suppress the strong forward fainting phenotype of the unc-80(ox330) mutant. (ns, P>0.05. Error bars = SEM; n = 36–38). (TIF) [file pgen.1007032.s008.tif]

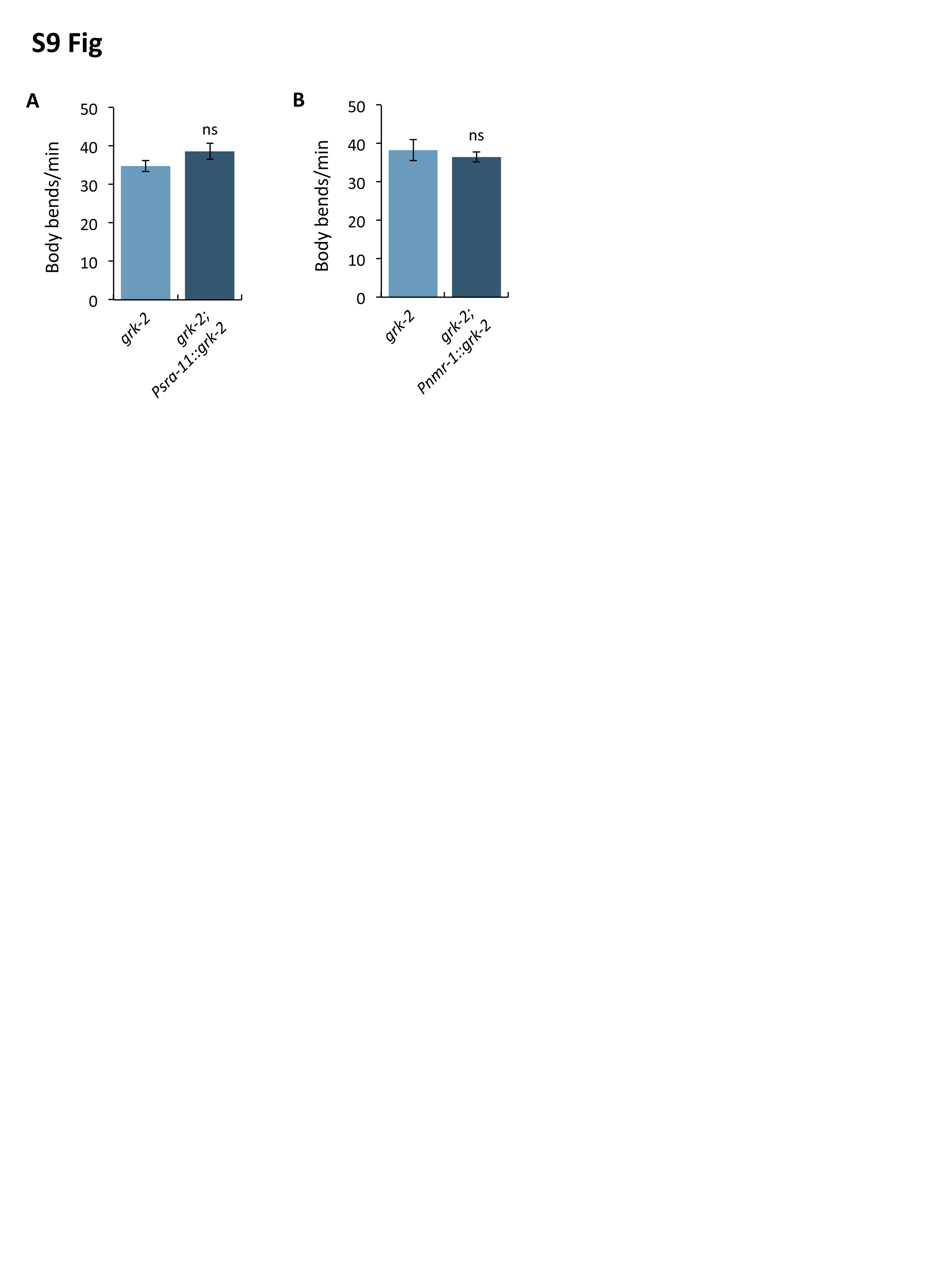

Supplement: S9 Fig — (A), (B) grk-2 cDNA expression driven by the (A) sra-11 (Psra-11, transgene yakEx147) or (B) nmr-1 (Pnmr-1, transgene yakEx85) promoter does not rescue the slow locomotion of the grk-2(gk268) mutant. (ns, P>0.05. Error bars = SEM; n = 10–20). (TIF) [file pgen.1007032.s009.tif]
