# Supplementary material for: Dopamine negatively modulates the NCA ion channels in C. elegans
Source: PLoS Genet. 2017 Oct 2;13(10):e1007032. doi: 10.1371/journal.pgen.1007032 (PMC5638609; doi:10.1371/journal.pgen.1007032)
Supplement: S1 Table — (DOCX) [file pgen.1007032.s010.docx]

**S1 Table. List of strains**

N2: Bristol wild strain

CB1112: *cat-2(e1112) II*

CB4856: Hawaiian wild strain

EG317: *unc-73(ox317) I*

EG330: *unc-80(ox330) V*

EG352: *nca-1(ox352) IV*

EG4782: *nzIs29[Punc-17::rho-1(G14V), unc-122::gfp] II*

EG5504: *nlf-1(tm3631) X*

FG7: *grk-2(gk268) III*

JT47: *egl-8(sa47) V*

EG3745: *eat-16(tm775) I ; him-5(e1490) V*

JT734: *goa-1(sa734) I*

LX645: *dop-1(vs100) X*

LX703: *dop-3(vs106) X*

LX705: *dop-1(vs100) X dop-3(vs106) X*

MT8504: *egl-10(md176) V*

PS2627: *dgk-1(sy428) X*

RB660: *arr-1(ok401)* X

XZ1151: *egl-30(tg26) I*

The following strains were produced in this study:

XZ18: *grk-2(yak18) III*

XZ1089: *egl-30(tg26) I ; grk-2(yak18) III*

XZ1527: *egl-30(tg26) I ; grk-2(gk268) III*

XZ1531: *grk-2(gk268) III ; egl-8(sa47) V*

XZ1532: *unc-73(ox317) I ; grk-2(gk268) III*

XZ1544: *grk-2(gk268) III ; yakEx44[Prab-3::grk-2 cDNA::tbb-2 3’UTR::OPERON::GFP::H2B, Pmyo-3::mCherry]*

XZ1549: *grk-2(gk268) III ; yakEx45[Punc-17::grk-2 cDNA::tbb-2 3’UTR::OPERON::GFP::H2B, Pmyo-2::mCherry]*

XZ1551: *grk-2(gk268) III ; yakEx47[Pacr-2::grk-2 cDNA::tbb-2 3’UTR::OPERON::GFP::H2B, Pmyo-2::mCherry]*

XZ1552: *grk-2(gk268) III ; yakEx48[GRK-2[K220R], Pmyo-2::mCherry]*

XZ1559: *nzIs29[Punc-17::rho-1(G14V), unc-122::gfp] II ; grk-2(gk268) III*

XZ1560: *grk-2(gk268) III ; nca-1(ox352) IV*

XZ1561: *grk-2(gk268) III ; yakEx51[Punc-17H::grk-2 cDNA::tbb-2 3’UTR::OPERON::GFP::H2B, Pmyo-3::mCherry]*

XZ1562: *grk-2(gk268) III ; yakEx52[Pglr-1::grk-2 cDNA::tbb-2 3’UTR::OPERON::GFP::H2B, Pmyo-2::mCherry]*

XZ1563: *grk-2(gk268) III ; yakEx53[Posm-6::grk-2 cDNA::tbb-2 3’UTR::OPERON::GFP::H2B, Pmyo-3::mCherry]*

XZ1571: *grk-2(gk268) III ; yakEx54[Pgrk-2::grk-2 cDNA::tagRFP, Pmyo-3::GFP]*

XZ1579: *grk-2(gk268) III ; yakEx55[GRK-2[Y109I], Pmyo-2::mCherry]*

XZ1581: *grk-2(gk268) III ; dgk-1(sy428) X*

XZ1582: *grk-2(gk268) III ; yakEx57[GRK-2[R106A], Pmyo-2::mCherry]*

XZ1583: *grk-2(gk268) III ; yakEx56[GRK-2[D110A], Pmyo-2::mCherry]*

XZ1641: *grk-2(gk268) III ; yakEx71[Pxbx-1::grk-2 cDNA::tbb-2 3’UTR::OPERON::GFP::H2B, Pmyo-2::mCherry]*

XZ1675: *grk-2(gk268) III ; yakEx77[GRK-2[D3K], Pmyo-2:mCherry]*

XZ1676: *grk-2(gk268) III ; yakEx78[GRK-2[L4K], Pmyo-2:mCherry]*

XZ1684: *eat-16(tm775) I ; grk-2(gk268) III*

XZ1691: *grk-2(gk268) III ; nlf-1(tm3631) X*

XZ1692: *grk-2(gk268) III ; yakEx79[GRK-2[V7A/L8A], Pmyo-2:mCherry]*

XZ1693: *grk-2(gk268) III ; yakEx80[GRK-2[D10A], Pmyo-2:mCherry]*

XZ1694: *grk-2(gk268) III ; dgk-1(sy428) X ; yakEx51[Punc-17H::grk-2 cDNA::tbb-2 3’UTR::OPERON::GFP::H2B,*

*Pmyo-3::mCherry]*

XZ1695: *grk-2(gk268) III ; dgk-1(sy428) X ; yakEx48[GRK-2[K220R], Pmyo-2::mCherry]*

XZ1713: *goa-1(sa734) I ; grk-2(gk268) III*

XZ1724: *grk-2(gk268) III ; yakEx85[Pnmr-1::grk-2cDNA::tbb-2 3’UTR::OPERON:GFP::H2B, Pmyo-2::mCherry]*

XZ1727: *grk-2(gk268) III ; yakEx87[GRK-2[K567E], Pmyo-2::mCherry]*

XZ1728: *grk-2(gk268) III ; yakEx88[GRK-2[R587Q], Pmyo-2::mCherry]*

XZ1729 *grk-2(gk268) III ; nca-1(gk9) IV*

XZ1730 *grk-2(gk268) III ; nca-2(gk5) III*

XZ1766: *grk-2(gk268) III ; yakEx95[GRK-2[R195A], Pmyo-2::mCherry]*

XZ1767: *grk-2(gk268) III ; yakIs19[Pgrk-2::grk-2 cDNA::tagRFP] ; yakEx94[Punc-17H::eGFP::let-858 3’UTR]*

XZ1845: *yakEx103[Punc-17H::GOA-1[Q205L]::tbb-2 3’UTR::OPERON::GFP::H2B, Pmyo-2::mCherry]*

XZ1859: *egl-10(md176) V ; nlf-1(tm3631) X*

XZ1876: *nlf-1(tm3631) X ; yakEx103[Punc-17H::GOA-1[Q205L]::tbb-2 3’UTR::OPERON::GFP::H2B, Pmyo-2::mCherry]*

XZ1903: *grk-2(gk268) III ; dop-3(vs106) X*

XZ1904: *egl-30(tg26) I ; grk-2(gk268) III ; dop-3(vs106) X*

XZ1905: *cat-2(e1112) II ; grk-2(gk268) III*

XZ1906: *egl-30(tg26) I ; cat-2(e1112) II ; grk-2(gk268) III*

XZ1909: *grk-2(gk268) III ; dop-3(vs106) X ; yakEx109[Pacr-2::dop-3 cDNA::tbb-2 3’UTR::OPERON::GFP::H2B,*

*Pmyo-2::mCherry]*

XZ1910: *grk-2(gk268) III ; dop-3(vs106) X ; yakEx110[Punc-17H::dop-3 cDNA::tbb-2 3’UTR::OPERON::GFP::H2B,*

*Pmyo-2::mCherry]*

XZ1911: *grk-2(gk268) III ; dop-3(vs106) X ; yakEx111[Punc-17::dop-3 cDNA::tbb-2 3’UTR::OPERON::GFP::H2B,*

*Pmyo-2::mCherry]*

XZ1912: *grk-2(gk268) III ; dop-3(vs106) X ; yakEx112[Prab-3::dop-3 cDNA::tbb-2 3’UTR::OPERON::GFP::H2B,*

*Pmyo-2::mCherry]*

XZ1925: *cat-2(e1112) II ; unc-80(ox330) V*

XZ1935: *cat-2(e1112) II ; nlf-1(tm3631) X*

XZ1936: *unc-80(ox330) V ; dop-3(vs106) X*

XZ1940: *nlf-1(tm3631) X dop-3(vs106) X*

XZ1941: *grk-2(gk268) III ; nlf-1(tm3631) X dop-3(vs106) X*

XZ2007: *grk-2(gk268) III ; dop-1(vs100) X dop-3(vs106) X*

XZ2028: *egl-30(tg26) I ; grk-2(gk268) III ; yakEx48[GRK-2(K220R), Pmyo-2::mCherry]*

XZ2029: *egl-30(tg26) I ; grk-2(gk268) III ; yakEx51[Punc-17H::grk-2 cDNA::tbb-2 3’UTR::OPERON::GFP::H2B,*

*Pmyo-3::mCherry]*

XZ2063: *dop-3(vs106) X ; yakEx130[Pgrk-2::dop-3 cDNA::GFP, Pmyo-2::mCherry]*

XZ2066: *nca-1(gk9) IV ; arr-1(ok401) X*

XZ2071: *grk-2(gk268) III ; yakEx135[Pcho-1(3.3 to 2.6)::grk-2 cDNA::tbb-2 3’UTR::OPERON::GFP::H2B, Pmyo-2::mCherry]*

XZ2075 *grk-2(gk268) III ; yakEx138[Pttx-3::grk-2 cDNA::tbb-2 3’UTR::OPERON::GFP::H2B, Pmyo-2::mCherry]*

XZ2078 *grk-2(gk268) III ; yakEx141[Psra-11::grk-2 cDNA::tbb-2 3’UTR::OPERON::GFP::H2B,*

*Pnmr-1::grk-2 cDNA:: tbb-2 3’UTR::OPERON::GFP::H2B, Pmyo-2::mCherry]*

XZ2086 *grk-2(gk268) III ; yakEx147[Psra-11::grk-2 cDNA::tbb-2 3’UTR::OPERON::GFP::H2B, Pmyo-2::mCherry]*

XZ2087 *grk-2(gk268) III ; dop-3(vs106) X ; yakEx148[Psra-11::dop-3 cDNA::tbb-2 3’UTR::OPERON::GFP::H2B,*

*Pnmr-1::dop-3 cDNA::tbb-2 3’UTR::OPERON::GFP::H2B, Pmyo-2::mCherry]*

XZ2088 *grk-2(gk268) III ; nlf-1(tm3631) X ; yakEx141[Psra-11::grk-2 cDNA::tbb-2 3’UTR::OPERON::GFP::H2B,*

*Pnmr-1::grk-2 cDNA:: tbb-2 3’UTR::OPERON::GFP::H2B, Pmyo-2::mCherry]*

XZ2089: *grk-2(gk268) III ; yakEx149[Pceh-24::grk-2 cDNA::tbb-2 3’UTR::OPERON::GFP::H2B, Pmyo-2::mCherry]*

XZ2090: *grk-2(gk268) III ; dop-3(vs106) X ; yakEx130[Pgrk-2::dop-3 cDNA::GFP, Pmyo-2::mCherry]*

XZ2091: *egl-30(tg26) I ; grk-2(gk268) III ; dop-3(vs106) X ; yakEx109[Pacr-2::dop-3 cDNA::tbb-2 3’UTR::OPERON::GFP::H2B, Pmyo-2::mCherry]*

XZ2095: *otIs534[Pcho-1^fosmid^::SL2::YFP::H2B] ; yakIs19[Pgrk-2::grk-2 cDNA::tagRFP]*
