# Supplementary material for: Dopamine negatively modulates the NCA ion channels in C. elegans
Source: PLoS Genet. 2017 Oct 2;13(10):e1007032. doi: 10.1371/journal.pgen.1007032 (PMC5638609; doi:10.1371/journal.pgen.1007032)
Supplement: S2 Table — (DOCX) [file pgen.1007032.s011.docx]

**S2 Table. List of plasmids**

Gateway destination vectors

pCFJ150 Gateway destination vector for insertion at chr II Mos site *ttTi5605*

Gateway entry clones

GL29 *Psra-11* [4-1] (4922 bp of the *sra-11* promoter upstream of the ATG)

pADA180 *Punc-17H* [4-1] (derived from the *unc-17* promoter of pGH1, carrying a 127 bp deletion that removes the nerve cord β enhancer, expressed in head acetylcholine neurons)

p_C06E1.4_93 *Pglr-1* [4-1] (from Open Biosystems) (727 bp of the *glr-1* promoter upstream of and including the ATG)

pCFJ31 *Pacr-2* [4-1] (3362 bp of the *acr-2* promoter upstream of the ATG)

pCFJ326  *tbb-2 3’UTR*::OPERON::GFP::H2B [2-3]

pCR185 GFP::*unc-54* 3’UTR [2-3]

pEGB05 *Prab-3* [4-1] (1210 bp of the *rab-3* promoter upstream of the ATG)

pET68 *grk-2* cDNA [1-2]

pET85 *Posm-6* [4-1] (2400 bp of the *osm-6* promoter upstream of the ATG)

pET89 *Pgrk-2* [4-1] (2895 bp of the *grk-2* promoter upstream of the ATG)

pET108 *Pxbx-1* [4-1] (373 bp of the *xbx-1* promoter upstream of the ATG)

pET134 *goa-1*[Q205L] cDNA [1-2]

pET139 *dop-3* cDNA with stop codon [1-2]

pET181 *dop-3* cDNA without stop codon [1-2]

pET185 *Pcho-1*(3.3-2.6) [4-1] (fragment of the *cho-1* promoter from 3300 bp to 2600 bp upstream of the ATG)

pET186 *Pceh-24* [4-1] (3013 bp of the *ceh-24* promoter upstream of the ATG)

pET196 *Pttx-3* [4-1] (414 bp fragment of the *ttx-3* genomic region starting from the second exon and including the AIY motif that directs expression only in AIY)

pGH1 *Punc-17* [4-1] (3229 bp of the *unc-17* promoter upstream of and including the ATG)

pGH107 tagRFP::*let-858* 3’UTR [2-3]

pMA102 *Pnmr-1* [4-1] (4709 bp of the *nmr-1* promoter upstream of the ATG)

Gateway expression constructs

pET79 *Prab-3::grk-2 cDNA::tbb-2 3’UTR*::OPERON::GFP::H2B_pCFJ150

pET81 *Punc-17::grk-2 cDNA::tbb-2 3’UTR:*:OPERON::GFP::H2B_pCFJ150

pET82 *Punc-17H::grk-2 cDNA::tbb-2 3’UTR*::OPERON::GFP::H2B_pCFJ150

pET83 *Pacr-2::grk-2 cDNA::tbb-2 3’UTR*::OPERON::GFP::H2B_pCFJ150

pET86 *Posm-6::grk-2 cDNA::tbb-2 3’UTR*::OPERON::GFP::H2B_pCFJ150

pET88 *Pglr-1::grk-2 cDNA::tbb-2 3’UTR*::OPERON::GFP::H2B_pCFJ150

pET90 *Pgrk-2::grk-2 cDNA*::GFP_pCFJ150

pET91 *Pgrk-2::grk-2 cDNA*::tagRFP_pCFJ150

pET93 *Punc-17H::eGFP::let-858 3’UTR*_pCFJ150

pET109 *Pxbx-1::grk-2 cDNA::tbb-2 3’UTR*::OPERON::GFP::H2B_pCFJ150

pET119 *Pnmr-1::grk-2 cDNA::tbb-2* *3’UTR*::OPERON::GFP::H2B_pCFJ150

pET135 *Punc-17H::GOA-1[Q205L]::tbb-2 3’UTR*::OPERON::GFP::H2B_pCFJ150

pET140 *Prab-3::dop-3 cDNA::tbb-2 3’UTR*::OPERON::GFP::H2B_pCFJ150

pET141 *Pacr-2::dop-3 cDNA::tbb-2 3’UTR*::OPERON::GFP::H2B_pCFJ150

pET142 *Punc-17::dop-3 cDNA::tbb-2 3’UTR*::OPERON::GFP::H2B_pCFJ150

pET143 *Punc-17H::dop-3 cDNA::tbb-2 3’UTR*::OPERON::GFP::H2B_pCFJ150

pET184 *Pgrk-2::dop-3 cDNA*::GFP_pCFJ150

pET190 *Pceh-24::grk-2 cDNA::tbb-2 3’UTR*::OPERON::GFP::H2B_pCFJ150

pET191 *Pcho-1(3.3-2.6)::grk-2 cDNA::tbb-2 3’UTR*::OPERON::GFP::H2B_pCFJ150

pET197 *Pttx-3::grk-2 cDNA::tbb-2* 3’UTR::OPERON-GFP::H2B_pCFJ150

pET209 *Pnmr-1::dop-3 cDNA::tbb-2 3’UTR*::OPERON::GFP::H2B_pCFJ150

pET214 *Psra-11::grk-2 cDNA::tbb-2 3’UTR*::OPERON::GFP::H2B_pCFJ150

pET215 *Psra-11::dop-3 cDNA::tbb-2 3’UTR*::OPERON::GFP::H2B_pCFJ150

Plasmids used for the GRK-2 structure-function analysis: from Wood et al., 2012.

pFG45 *Pgrk-2::*GRK-2

pFG46 *Pgrk-2::*GRK-2[D3K]

pFG47 *Pgrk-2::*GRK-2[L4K]

pFG48 *Pgrk-2::*GRK-2[V7A/L8A]

pFG49 *Pgrk-2::*GRK-2[D10A]

pFG84 *Pgrk-2::*GRK-2[R195A]

pFG85 *Pgrk-2::*GRK-2[R106A]

pFG86 *Pgrk-2::*GRK-2[Y109I]

pFG87 *Pgrk-2::*GRK-2[D110A]

pFG88 *Pgrk-2::*GRK-2[K220R]

pFG89 *Pgrk-2::*GRK-2[K567E]

pFG90 *Pgrk-2::*GRK-2[R587Q]
